# Insight into ferromagnetic interactions in Cu^II^–Ln^III^ dimers with a compartmental ligand†

**DOI:** 10.1039/d3dt03557c

**Published:** 2023-12-27

**Authors:** Anangamohan Panja, Sagar Paul, Eufemio Moreno-Pineda, Radovan Herchel, Narayan Ch. Jana, Paula Brandão, Ghenadie Novitchi, Wolfgang Wernsdorfer

**Affiliations:** a Department of Chemistry, Gokhale Memorial Girls’ College 1/1 Harish Mukherjee Road Kolkata-700020 India ampanja@yahoo.co.in; b Department of Chemistry, Panskura Banamali College Panskura RS WB 721152 India; c Physikalisches Institut, Karlsruhe Institute of Technology D-76131 Karlsruhe Germany wolfgang.wernsdorfer@kit.edu; d Universidad de Panamá, Facultad de Ciencias Naturales, Exactas y Tecnología, Depto. de Química-Física 0824 Panamá Panama; e Universidad de Panamá, Facultad de Ciencias Naturales, Exactas y Tecnología, Grupo de Investigación de Materiales 0824 Panamá Panama; f Department of Inorganic Chemistry, Faculty of Science, Palacký University 17. listopadu 12 77146 Olomouc Czech Republic; g Department of Chemistry, CICECO-Aveiro Institute of Materials, University of Aveiro 3810-193 Aveiro Portugal; h Laboratoire National des Champs Magnétiques Intenses, UPR CNRS 3228, Université Grenoble-Alpes B.P. 166 38042 Grenoble Cedex 9 France; i Institute for Quantum Materials and Technology (IQMT), Karlsruhe Institute of Technology (KIT) Eggenstein-Leopoldshafen D-76344 Germany

## Abstract

In the last two decades, efforts have been devoted to obtaining insight into the magnetic interactions between Cu^II^ and Ln^III^ utilizing experimental and theoretical means. Experimentally, it has been observed that the exchange coupling (*J*) in Cu^II^–Ln^III^ systems is often found to be ferromagnetic for ≥4f^7^ metal ions. However, exchange interactions at sub-Kelvin temperatures between Cu^II^ and the anisotropic/isotropic Ln^III^ ions are not often explored. In this report, we have synthesized a series of heterobimetallic [CuLn(HL)(μ-piv)(piv)_2_] complexes (Ln^III^ = Gd (1), Tb (2), Dy (3) and Er (4)) from a new compartmental Schiff base ligand, *N*,*N’*-bis(3-methoxy-5-methylsalicylidene)-1,3-diamino-2-propanol (H_3_L). X-ray crystallographic analysis reveals that all four complexes are isostructural and isomorphous. Magnetic susceptibility measurements reveal a ferromagnetic coupling between the Cu^II^ ion and its respective Ln^III^ ion for all the complexes, as often observed. Moreover, μ-SQUID studies, at sub-Kelvin temperatures, show S-shaped hysteresis loops indicating the presence of antiferromagnetic coupling in complexes 1–3. The antiferromagnetic interaction is explained by considering the shortest Cu⋯Cu distance in the crystal structure. The nearly closed loops for 1–3 highlight their fast relaxation characteristics, while the opened loops for 4 might arise from intermolecular ordering. CASSCF calculations allow the quantitative assessment of the interactions, which are further supported by BS-DFT calculations.

## Abstract 2

A series of heterobimetallic ferromagnetically coupled Cu^II^-Ln^III^ systems with a new compartmental Schiff base ligand has been explored by SQUID and μ-SQUID studies. CASSCF calculations allow quantitative assessment of the interactions, which are further supported by BS-DFT calculations.

## Graphical abstract

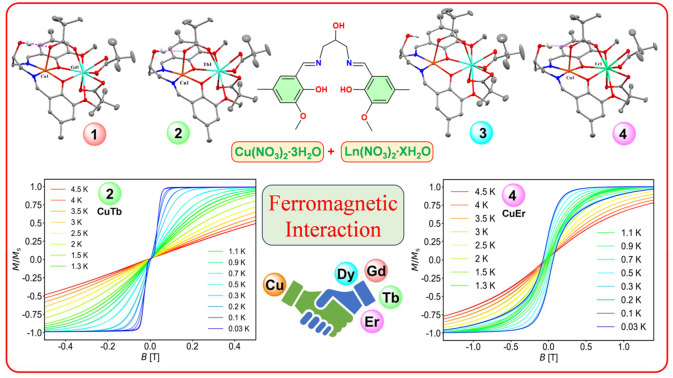

## Introduction

Heterometallic complexes with 3d and 4f metal ions have considerably attracted the interest of researchers due to their numerous advantages in the fields of catalysis,^1^ luminescence^2,3^ and molecular magnetism.^4–6^ In the field of molecular magnetism, their large magnetic moment and strong single-ion anisotropy make 4f ions appropriate candidates for the development of Single-Molecule Magnets (SMMs), a class of compounds usually characterized by their high-spin ground state, slow relaxation of magnetization and large axial anisotropy.^7–11^ A relatively strong magnetic exchange interaction between 3d and 4f metal ions, compared to those between 4f centres, yields further tunability of such magnetic characteristics in the 3d–4f cages. In several 3d–4f compounds, the exchange interaction between 3d and 4f ions has been found to effectively suppress the fast zero-field Quantum Tunnelling of Magnetization (QTM) of lanthanide ions, which in turn enhances the SMM behaviour.^12–14^ In general, the magnetic interaction between transition-metal and lanthanide ions plays a crucial role in defining the magnetic behaviour and applicability of such magnetic materials and hence needs to be evaluated. The pioneering work in the field of 3d–4f systems by Gatteschi *et al*. revealed a fairly strong Cu–Gd ferromagnetic exchange interaction,^15^ which was found to be an ideal model to study the magnetic exchange between transition metals and lanthanide ions owing to the isotropic nature of the Gd^III^ ion.^16,17^ Subsequent studies in this field further ascertain the usual ferromagnetic coupling between Cu^II^ and Gd^III^ ions in the synthesized compounds in which the degree of planarity of the bridging core plays a crucial role in the magnetic exchange coupling between the two metal ions.^18–21^ Furthermore, Matsumoto and co-workers successfully synthesized the first 3d–4f SMM complex [Cu^II^Tb^III^L^1^(hfac)_2_]_2_ (H_3_L^1^ = 1-(2-hydroxybenzamido)-2-(2-hydroxy-3-methoxy-benzylideneamino)-ethane), in which the magnetic interaction between Cu^II^ and Tb^III^ ions has been proven to be critical for its SMM behaviour,^22^ and thereafter, the 3d–4f system is found to be increasingly important owing to its promising applications in quantum storage and spintronic devices.^23–28^

**Scheme 1:**
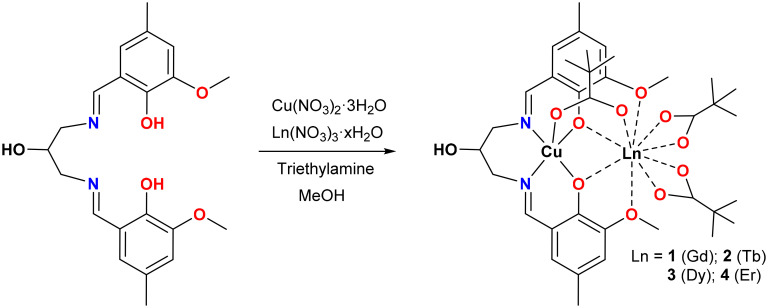
Schematic synthetic route for complexes 1–4.

The preparation of complexes containing only a limited number of 3d and 4f ions is crucial, particularly when the study is focused on gaining an in-depth understanding of the 3d–4f magnetic interactions. In such a case, the automatic choice is a simple heterobimetallic 3d–4f complex, well isolated from its congeners. Among several ligand systems, classical salen-type compartmental Schiff base ligands derived from *o*-vanillin and diamines are extensively utilized for the construction of heteronuclear 3d–4f complexes, because they offer two distinct preorganised coordination pockets to selectively accommodate 3d metal ions through a smaller and inner compartment with N_2_O_2_ donor sites and the lanthanide ions through a larger and outer compartment with O_2_O′_2_ donor sites.^29–32^ Several 3d–4f complex SMMs have also been reported to date using this class of compartmental ligands in which the ferromagnetic interaction between the metal ions is found to be significant.^33–42^ We have been working with new members of Schiff base ligands in the widespread family of salen-type compartmental Schiff base ligand systems derived from a methyl substituted *o*-vanillin to explore 4f or 3d–4f-based SMMs.^43–46^ As a follow-up, in the present work, we intend to develop Cu–Ln-based complexes with another new member of the salen-type compartmental ligand family, *N*,*N’*-bis(3-methoxy-5-methylsalicylidene)-1,3-diamino-2-propanol (H_3_L) (Scheme 1). Among the heavier Ln^III^ ions, the obvious choice is the Dy^III^ ion as the vast majority of reported SMMs are based on this oblate Kramer's ion together with the next most studied systems with oblate and non-Kramer's characteristics such as the Tb^III^ ion and Kramer's Er^III^ ion with prolate electron distribution.^47–50^ Moreover, the isotropic Gd^III^ ion has also been synthesized to obtain precise information regarding the magnetic exchange between the spin carriers in this system.^51–53^ Unlike Ni^II^ and Co^II^ ions,^54,55^ the isotropic Cu^II^ ion does not contribute to the magnetic anisotropy at the molecular level, but the usual ferromagnetic coupling between Cu^II^ and heavier lanthanide ions (4f^7^ onwards), irrespective of the nature of binding and nuclearity of the systems, makes it useful in studying the magnetic behaviour in 3d–4f compounds.^56–61^ Keeping all these in mind, the ligand H_3_L was allowed to react with respective hydrated lanthanide nitrates, Ln(NO_3_)_3_·*x*H_2_O (*x* = 5 or 6), in the presence of pivalic acid and a triethylamine base to afford isostructural and isomorphous [CuLn(HL)(μ-piv)(piv)_2_] complexes (Ln^III^ = Gd (1), Tb (2), Dy (3) and Er (4)). Herein, we report the synthesis, structures and low temperature and sub-Kelvin magnetic properties of the system *via* SQUID and μ-SQUID studies, and CASSCF and BS-DFT calculations were further performed to interpret the experimental results in these complexes.

## Experimental section

### Materials and methods

Reagent or analytical grade chemicals and solvents used in the present study were purchased from commercial sources and used without further purification. 2-Hydroxy-3-methoxy-5-methylbenzaldehyde (Me-val) was synthesized following a literature reported method.^62^ All reactions were carried out under aerobic conditions.

### Synthesis of complexes 1–4

All four binuclear [CuLn(HL)(μ-piv)(piv)_2_] complexes (Ln^III^ = Gd (1), Tb (2), Dy (3) and Er(4)) have been synthesized by one-pot reactions and in a step-wise manner without the isolation of an intermediate Cu^II^ complex. The synthetic procedure consists of *in situ* formation of the Schiff-base ligand H_3_L by the condensation reaction of Me-val (0.50 mmol) and 1,3-diamino-2-propanol (0.25 mmol) in methanol under continuous stirring for about 30 minutes, followed by the addition of triethylamine (0.50 mmol) and Cu(NO_3_)_2_·3H_2_O (0.25 mmol), and the stirring was continued for another 1 hour. Then a methanolic solution (10 mL) of the corresponding hydrated lanthanide nitrate, Ln(NO_3_)_3_·6H_2_O (Ln^III^ = Gd) or Ln(NO_3_)_3_·5H_2_O (Ln^III^ = Tb, Dy and Er) (0.25 mmol), pivalic acid (piv, 0.75 mol) and triethylamine (0.75 mmol) were added to the reaction mixture. The mixture was continuously stirred for another 1 hour. The resulting dark green solution was then filtered and left undisturbed for slow evaporation at ambient temperature. After a week, X-ray quality dark green single crystals of complexes 1–4 were obtained. The crystals were collected by filtration, washed with methanol, and dried in air.

#### [CuGd(HL)(μ-piv)(piv)_2_] (1)

Yield: 191 mg (84%). Anal. calcd for C_36_H_51_CuGdN_2_O_11_: C 47.59%, H 5.66%, N 3.08%. Found: C 47.77%, H 3.75%, N 2.94%.

#### [CuTb(HL)(μ-piv)(piv)_2_] (2)

Yield: 180 mg (79%). Anal. calcd for C_36_H_51_CuTbN_2_O_11_: C 47.50%, H 5.65%, N 3.08%. Found: C 47.66%, H 3.71%, N 2.84%.

#### [CuDy(HL)(μ-piv)(piv)_2_] (3)

Yield: 196 mg (86%). Anal. calcd for C_36_H_51_CuDyN_2_O_11_: C 47.32%, H 5.62%, N 3.07%. Found: C 47.69%, H 3.72%, N 2.86%.

#### [CuEr(HL)(μ-piv)(piv)_2_] (4)

Yield: 200 mg (87%). Anal. calcd for C_36_H_51_CuErN_2_O_11_: C 47.07%, H 5.60%, N 3.05%. Found: C 47.26%, H 3.69%, N 2.89%.

### Physical measurements

Elemental analyses (C, H, N) were conducted using a PerkinElmer 240C elemental analyser. FT-IR spectra were recorded using a Thermo Scientific Nicolet iS5 FTIR Spectrometer using a universal ATR sampling accessory from 4000 to 400 cm^−1^.

### X-ray crystallography

The single crystal X-ray diffraction study of complexes 1–4 was carried out on a Bruker Smart Apex-II CCD diffractometer with monochromated Mo-Kα radiation (*λ* = 0.71073 Å) at 150 K. Several scans were obtained in the *φ* and *ω* directions to increase the number of redundant reflections and were averaged during the refinement cycles. The intensity data were processed using the Bruker APEX-II suite (v2.0-2) program, then absorption correction and inter-frame scaling were performed and other systematic errors were fixed using the SADABS program.^63^ All structures were resolved by direct methods with SHELXT 2014/5 and then refined using the full matrix least-squares technique based on *F*^2^ using SHELXL2018/3.^64^ All non-hydrogen atoms were refined with anisotropic thermal displacement parameters. Hydrogen atoms connected to carbon atoms were placed at geometrically idealized positions with individual isotropic thermal parameters equal to 1.2 or 1.5 times the riding atoms, while the proton of the alcoholic–OH group was located in the difference Fourier map and refined freely. The molecular view and packing diagrams of the complexes were obtained using the Mercury-4.3.1 and POV-ray software. A summary of crystallographic data and refinement details is available in Table S1.†

### CASSCF calculations

The *ab initio* calculations for all complexes were performed employing the complete active space self-consistent field (CASSCF) method, specifically the CASSCF/SO-RASSI/SINGLE_ANISO approach implemented in the OpenMolcas package.^65^ For the calculations, the crystal structures were used as obtained from the single crystal X-ray analysis without further optimization. All atoms were described with the standard basis sets from the ANO-RCC library.^66–68^ For the anisotropic lanthanides, a basis set of VTZP quality was employed, while VDZP quality was employed for atoms in the first coordination sphere of the lanthanide. Basis sets of VDZ quality were used for the remaining atoms. Optimization of the molecular orbitals (MOs) was achieved by state averaged CASSCF calculations. The Tb^III^ active space was defined by eight 4f electrons in the seven 4f orbitals. Four calculations were carried out (the RASSCF routine) with 7, 140, 588 and 490 states for *S* = 3, *S* = 2, *S* = 1 and *S* = 0, respectively. The CASSCF wavefunctions were subsequently mixed by spin–orbit coupling, employing the RASSI routine with all 7 states for *S* = 3 being included, while 106, 283 and 121 states were included for *S* = 2, *S* = 1 and *S* = 0, respectively. Lastly, the crystal field decomposition of the ground *J* = 6 multiplet of the ^7^F_6_ term was executed with the SINGLE_ANISO module. For Dy^III^, the active space was defined by the nine 4f electrons in the seven 4f orbitals. The RASSCF routine was carried out with 21, 224 and 490 states for *S* = 5/2, *S* = 3/2 and *S* = ½, respectively. The RASSI^69^ routine was executed for all 21 states for *S* = 5/2 being included, while 128 and 130 states were included for *S* = 3/2 and *S* = ½, respectively, and the crystal field decomposition of the ground *J* = 15/2 multiplet of the ^6^H_15/2_ term was executed with SINGLE_ANISO. The Er^III^ active space was defined by the eleven 4f electrons in the seven 4f orbitals. Two RASSCF calculations were conducted in this case with 35 and 112 states for *S* = 3/2 and *S* = ½, respectively. The RASSI routine was executed for all 35 states for *S* = 3/2 and 112 for the *S* = ½ state. The crystal field decomposition of the ground *J* = 15/2 multiplet of the ^4^I_15/2_ term was likewise executed with SINGLE_ANISO.^70,71^

### DFT calculations

Density Functional Theory (DFT) calculations were performed using ORCA 5.0 software.^72^ The ZORA relativistic approximation was used,^73^ together with ZORA-def2-TZVPP for Cu and Zn atoms, SARC2-ZORA-QZVP for lanthanide atoms, ZORA-def2-TZVP for N and O atoms, and ZORA-def2-SVP for C and H atoms.^74,75^ The calculations were sped up using the SARC/J Coulomb fitting basis set^76^ and the RIJCOSX approximation.^77^ The largest integration grid (DEFGrid3) and tightSCF convergence criteria were used in all calculations. The molecular structures were extracted from the X-ray data, and the atomic positions of the hydrogen atoms were optimized with the BP86 functional^78^ incorporating atom-pairwise dispersion correction (D4).^79^ The calculated data were visualized with the VESTA 3 program.^80^

### Magnetic measurements

The magnetic susceptibility studies for all the complexes were performed on a Quantum Design MPMS®3 SQUID magnetometer employing polycrystalline materials. The temperature range of the study was 2–300 K under an applied DC magnetic field of 1 kOe. AC data were collected using an oscillating magnetic field of 3.5 Oe and frequencies between 1 and 1.5 kHz. The DC data were corrected for diamagnetic contributions from the eicosane and core diamagnetism. The low-temperature magnetization measurements (0.03–5 K) were performed on single crystals *via* μ-SQUID apparatus at different sweep rates between 0.001 and 0.128 T s^−1^ with a time resolution of approximately 1 ms. The magnetic field can be applied in any direction of the μ-SQUID plane with a precision better than 0.1° by separately driving three orthogonal coils. The magnetic field was applied parallel to the easy axis of magnetization found using the transverse field method. To ensure good thermalization, each sample was fixed with apiezon grease.

## Results and discussion

### Synthesis and characterization of Cu–Ln complexes

The salen-type compartmental Schiff base ligand derived from *o*-vanillin has been extensively utilized for the construction of heterometallic 3d–4f complexes with intriguing magnetic properties. In the present work, a new member of the salen-type compartmental Schiff base ligand H_3_L was prepared by the condensation reaction of Me-val and 1,3-diamino-2-propanol in a 2 : 1 ratio in methanol. This *in situ* generated ligand H_3_L thereafter was allowed to react with Cu(NO_3_)_2_·3H_2_O and the corresponding hydrated lanthanide nitrate, Ln(NO_3_)_3_·6H_2_O (Ln^III^ = Gd) or Ln(NO_3_)_3_·5H_2_O (Ln^III^ = Tb, Dy and Er), in 1 : 1 : 1 molar ratio in the presence of 3 equivalents of pivalic acid to afford dark green crystals of [CuLn(HL)(μ-piv)(piv)_2_] (Ln^III^ = Gd (1), Tb (2), Dy (3) and Er (4)) in high yield. The triethylamine base was added to deprotonate both ligand H_3_L and pivalic acid during the course of the reaction. In the IR spectra of the complexes (Fig. S1†), a broad band at ∼3280 cm^−1^ can be assigned to the *ν*(OH) vibration of the pendent hydroxyl group of the ligand, and a broad and bifurcated band at 1611–1629 cm^−1^ is consistent with the overlapping vibrations of the imine bond of the Schiff base and pivalate ion. Additionally, powder X-ray diffraction measurements were employed to confirm the phase purity of all the complexes (Fig. S2†).

### Structural descriptions

**Fig. 1:**
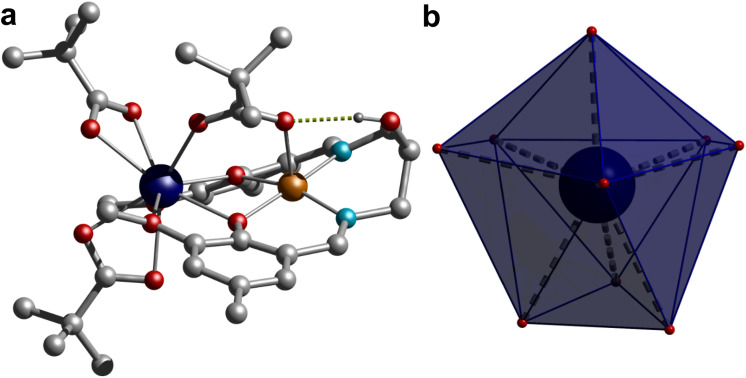
(**a**) Single crystal X-ray structure of complex 1 and hydrogen bonded proton to the carboxylate and (**b**) local coordination geometry around the Gd^III^ centre. Colour code: Gd, dark blue; Cu, orange; N, cyan; C, grey; O, red; H, dark grey. Hydrogen atoms on pivalates and ligands were omitted for clarity.

Single crystal structure analysis of the synthesized complexes reveals that all these heterobimetallic Cu^II^Ln^III^ complexes are isostructural and isomorphous, crystallized in the triclinic *P*1̄ space group. The crystal structure of 3 is shown in Fig. 1, while the rest are displayed in Fig. S3,† and the selected bond distances are given in Table S2.† The asymmetric unit contains a neutral complex molecule with a general formula [CuLn(HL)(μ-piv)(piv)_2_], in which the Cu^II^ and Ln^III^ ions occupy the inner N_2_O_2_ and outer O_2_O′_2_ compartments of the doubly deprotonated Schiff base ligand, respectively. Additionally, a pivalate ion bridges the metal centres in a κO:κO′ bridging fashion and two remaining pivalate ions bind the Ln^III^ ions as a bidentate chelating ligand (κ^2^O,O′), resulting in CuN_2_O_3_ and LnO_9_ coordination environments around the Cu^II^ and Ln^III^ ions, respectively, in these complexes. The Cu^II^ ion is invariably five-coordinated with a square-pyramidal geometry in all four complexes, in which the metal centre is slightly displaced (∼0.167 Å) from the N_2_O_2_ least squares plane towards the apically coordinated bridging pivalate-O atom, typical of a square pyramidal geometry, which is further supported by extracted continuous-shape measure (CShM) values on the basis crystallographic coordinates around the Cu^II^ ion using the Shape 2.1 software [Table S3†]. The Cu–O and Cu–N bond distances spanning the range 1.9373(10)–2.2164(16) Å are usual for Cu^II^ complexes.

The geometry of the nona-coordinated Ln^III^ ions in these complexes was also analysed using CShMs implemented in the Shape 2.1 software using the crystallographic coordinates, which reveals that the geometry of the Ln^III^ ions is in-between a muffin-like geometry with *C*_s_ symmetry and a spherical capped square antiprism geometry with *C*_4v_ symmetry as suggested by the CShM values given in Table S4.† The Ln–O bond distances with the phenolate–O atom and the bridging pivalate ion are shorter and span the range 2.2929(10)–2.4021(15) Å, while the Ln–O bond distances of the methoxy group are significantly longer and vary in the range 2.4745(10)–2.6525(15) Å. The Ln–O bond distances of the chelating pivalate groups fall in the intermediate range, spanning between 2.3502(10) and 2.4720(16) Å. Moreover, a typical consequence of the Ln^III^ contraction has been observed as the respective Ln–O bond distances in these systems follow the order Gd > Tb > Dy > Er. Careful inspection of crystal packing reveals that the adjacent complex molecules interact with each other by the π⋯π interaction (Fig. S4†). Moreover, the pendent hydroxyl group of the ligand establishes an intramolecular hydrogen bond with the bridging pivalate ion. The CuO_2_Ln core is not planar as suggested by the dihedral angle of about 24.45° between the CuO_2_ and GdO_2_ planes in these complexes. The bridging pivalate ligand together with the aforesaid hydrogen bonding interaction involving the aliphatic hydroxyl group of the ligand and the bridging pivalate group is mainly responsible for the deviation of the CuO_2_Ln core from planarity. The Ln–O–Cu bridging angles vary in the narrow range of 103.29(14)–105.88(10)° with intermolecular Cu⋯Ln separations of 3.3758(3)–3.4206(3) Å, while the closest intermolecular Ln⋯Ln, Cu⋯Ln and Cu⋯Cu separations vary in the range 7.589–7.646, 7.206–7.321 and 6.109–6.343 Å, respectively, in these complexes.

### Magnetic studies

**Fig. 2:**
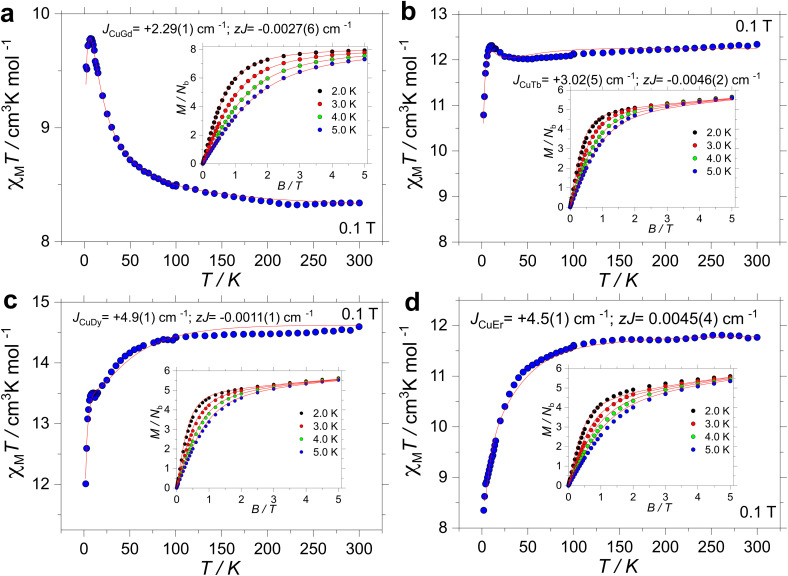
Experimental *χ*_M_*T*(*T*) and *M*(*B*, *T*) (insets) for complex 1 (**a**), 2 (**b**), 3 (**c**) and 4 (**d**) under an applied field of 1 kOe. The red lines are the fits as described in the text.

High-temperature SQUID measurements were conducted on powdered samples. The observed *χ*_M_*T* values were found to be 8.34, 12.34, 14.59 and 11.76 cm^3^ K mol^−1^ for 1, 2, 3 and 4, respectively (Fig. 2). All room temperature *χ*_M_*T* values are found to be in agreement with the expected values for the isolated ions (*cf.*, 8.25 cm^3^ K mol^−1^ for 1 with *S* = ½ and *g* = 2.0 for one Cu^II^ and *S* = 7/2 and *g* = 2 for one Gd^III^; 12.19 cm^3^ K mol^−1^ for 2 with *S* = ½ and *g* = 2.0 for one Cu^II^ and *J* = 6 and *g*_*J*_ = 3/2 for one Tb^III^; 14.54 cm^3^ K mol^−1^ for 3 with *S* = ½ and *g* = 2.0 for one Cu^II^ and *J* = 15/2 and *g*_*J*_ = 4/3 for one Dy^III^ and 11.85 cm^3^ K mol^−1^ for 4 with *S* = ½ and *g* = 2.0 for one Cu^II^ and *J* = 15/2 and *g*_*J*_ = 6/5 for one Er^III^). Upon cooling, *χ*_M_*T*(*T*) is contingent on the lanthanide ion. For 1, however, below 200 K, *χ*_M_*T* increases smoothly down to ∼50 K, and it sharply increases to a maximum value of 9.78 cm^3^ K mol^−1^ at 7 K. For 2, *χ*_M_*T*(*T*) stays nearly constant down to 25 K, and it increases up to 12.31 cm^3^ K mol^−1^ at 10 K. For 3, *χ*_M_*T*(*T*) decreases smoothly down to around 50 K, and it drops faster before increasing slightly between 12 and 6 K. The upsurge renders a *χ*_M_*T*(*T*) value of 13.50 cm^3^ K mol^−1^ at 10 K, before decreasing again down to a minimum value of 12.01 cm^3^ K mol^−1^ at 2.0 K. Finally, the *χ*_M_*T*(*T*) profile for 4 decreases smoothly down to ∼50 K, and it drops faster reaching a minimum value of 8.35 cm^3^ K mol^−1^ at 2.0 K. The observed upsurge in complexes 1–3 is characteristic of ferromagnetic interactions, often observed in 3d/4f complexes.^33,37,81,82^

Magnetization *versus* field studies were likewise conducted for all complexes, leading to values of 7.92, 5.65, 5.63 and 5.60*N*_*β*_ for 1, 2, 3 and 4, respectively. Note that saturation of *M*(*B*, *T*) was observed only for 1, while for 2–4, *M*(*B*, *T*) highlights anisotropy of the systems.

The isotropic nature of complex 1 allows us to simultaneously fit^83^ the *χ*_M_*T*(*T*) and *M*(*B*, *T*) data employing an isotropic exchange interaction operating between the Cu^II^ and Gd^III^ ions. The fitting procedure was carried out employing PHI.^83^ The Hamiltonian, hence, has the following form:

1 *Ĥ* = −2*JŜ*_Gd_·*Ŝ*_Cu_ + *μ*_B_*B*(*g*_Gd_*Ŝ*_Gd_ + *g*_Cu_*Ŝ*_Cu_)

where the first term represents the interaction between the Cu^II^ and Gd^III^ centres and the second one is the Zeeman term. Simultaneous fitting of *χ*_M_*T*(*T*) and *M*(*B*, *T*), varying solely *J*, while fixing *g*_Cu_ = *g*_Gd_ = 2.0, yields a ferromagnetic interaction *J* = +2.29(1) cm^−1^, as often observed in 3d–4f complexes.^52,81,82,84^ For the fitting, a weak interchain interaction was also employed *zJ* = −0.0027(6) cm^−1^. The intramolecular interaction is ferromagnetic, in line with other reported Cu⋯Gd pairs.^33,37,53,85^ At this stage, no further analysis is possible for the complexes containing anisotropic lanthanides; however, from the *χ*_M_*T*(*T*) profile, ferromagnetic interactions are also expected for 2 and 3.

To probe the Single Molecule Magnet (SMM) characteristic of the anisotropic complexes, AC studies were conducted. Nonetheless, no frequency-dependent AC signal was detected for the complexes in the temperature range of the study at zero field or upon application of an *H*_DC_ field, and hence, none of the complexes is an SMM.

#### μ-SQUID measurements

To further investigate the complexes, magnetic measurements on single microcrystals of the four Cu–Ln molecules were performed using a μ-SQUID setup. The single microcrystals were placed in the vicinity of an array of μ-SQUIDs and thermalized using apiezon grease. From the array, a μ-SQUID with optimum coupling to the crystal was selected for the measurements. A 3D vector magnet was used to control and align the field direction concerning the crystal. The ‘transverse field method’ was used to find the easy axis of the crystal, along which the temperature and field sweep-rate dependent *M*(*B*) loops were studied.^86^

**Fig. 3:**
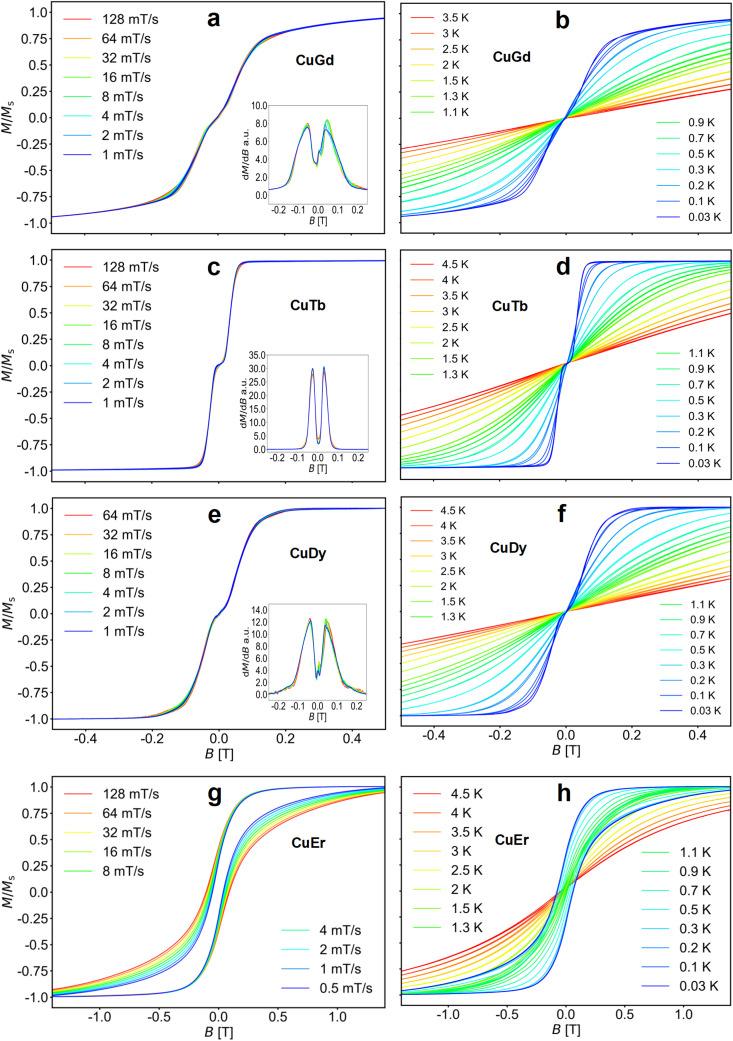
(**a**, **c**, **e** and **g**) Field sweep dependent M–B loops for CuLn molecules (Ln = Gd, Tb, Dy, and Er, respectively) obtained at *T* = 30 mK. Insets of (**a**), (**c**) and (**e**) show the derivatives dM/dB *vs*. *B* indicating the exchange fields. (**b**, **d**, **f** and **h**) Corresponding M–B loops obtained at different temperatures at a fixed sweep rate of 8 mT s^−1^.

Fig. 3 (left panel) shows the *M*(*B*) curves obtained for a single crystal at 30 mK temperature and different sweep rates of the magnetic field, while the right panel shows the temperature dependence of the *M*(*B*) loops up to about 4.5 K.

The loops for complexes 1–3 are nearly closed, highlighting fast relaxation. Moreover, the sweep rate independent transitions indicate QTM. Note that samples 1, 2 and 3 show a flat *M*(*B*) about zero field (S-shaped loops), a signature of antiferromagnetic coupling.^86–89^ This is clearly observed in the derivatives shown in the insets of Fig. 3. Upon increasing the temperature, the QTM transitions broaden, while the small hysteresis opening diminishes. At 100 mK bath temperature and above, the antiferromagnetic plateau near zero field in 1–3 is averaged out or disappears. In contrast, the loops for 4 are open up to ∼1 K, indicating slower relaxation.

As observed in the SQUID studies, 3d metals typically couple to the neighbouring Ln ferromagnetically,^37,81,82^ hence the antiferromagnetic arrangement could be of inter-molecular origin. Considering the inner nature of 4f orbitals and the shortest Ln⋯Ln and Cu⋯Cu distances, the most probable intermolecular interaction is between Cu⋯Cu intermolecular contacts. To assess the strength of the interactions in complexes 1–3, we directly extract the interaction from the μ-SQUID *M*(*B*) loops through the mean exchange field *H*_ex_ = −2*J*_ex_*m*_*S*_/*gμ*_B_, where *J*_ex_ is the interaction, *m*_*S*_ is the spin for Cu^II^ (*S* = ½), *g* = 2.0 and *μ*_B_ is the Bohr magneton. Considering that the crossing for 1 occurs at ±0.101 T, *J*_ex_ = −0.38 cm^−1^. For 2 and 3, *J*_ex_ values of −0.22 and −0.31 cm^−1^ were obtained, for the crossings at ±0.058 and ±0.082 T, respectively. Considering the shortest Cu⋯Cu distance in the crystal structure, we find that the strongest dipolar interaction is −0.034 cm^−1^, which is ten times smaller than the *J*_ex_, hence, the intermolecular exchange might be operative. Due to the lack of S-shaped loops in 4, no *J*_ex_ can be determined for this complex.

### Theoretical calculations

To understand the magnetic characteristics of studied compounds, DFT calculations were performed for 1–4 with ORCA, and more advanced *ab initio* calculations for 2–4 employing OpenMolcas (see methods section) were carried out.

First, the isotropic exchange parameter *J* (using definition *Ĥ* = −2*J*(*Ŝ*_1_·*Ŝ*_2_)) was evaluated using the broken-symmetry DFT calculations by comparing the energies of high-spin (HS) and broken-symmetry (BS) spin states, *Δ* = *ε*_BS_ − *ε*_HS_, with two approaches defined by Ruiz and Yamaguchi as:

2 *J*^R^ = 2*Δ*/[(*S*_1_ + *S*_2_)(*S*_1_ + *S*_2_ + 1)]

3 *J*^Y^ = 2*Δ*/[*S*^2^_HS_ − *S*^2^_BS_]

Herein, three different DFT functionals were used: a hybrid functional PBE0 and hybrid *meta*-GGA functionals M06-2X and TPSSh. Such functionals are among those usually applied for the calculations of magnetic exchange for 3d–3d/3d–4f/4f–4f complexes.^90–93^ First, intramolecular interactions were evaluated for all Cu–Ln complexes, as summarized in Table 1, and the BS-DFT spin densities are shown in Fig. S5.†

**Table 1:** The BS-DFT calculations of the intramolecular isotropic exchange parameters *J*_CuLn_ for 1–4

	*J* ^R^/*J*^Y^ (cm^−1^) of Cu–Gd in 1	*J* ^R^/*J*^Y^ (cm^−1^) of Cu–Tb in 2	*J* ^R^/*J*^Y^ (cm^−1^) of Cu–Dy in 3	*J* ^R^/*J*^Y^ (cm^−1^) of Cu–Er in 4
PBE0	0.81/2.32	0.93/2.44	1.68/4.03	3.69/7.38
M06-2X	0.69/1.97	0.75/1.96	0.74/1.78	0.83/1.67
TPSSh	0.75/2.15	1.21/3.18	4.78/11.48	1.25/2.49

Evidently, all three DFT functionals suggest ferromagnetic exchange in the presented complexes, which is in accordance with the analysis of the experimental magnetic data. The closest match to the fitted value *J*_CuGd_ = 2.29 cm^−1^ is found for the PBE0 functional (*J*^Y^ = 2.32 cm^−1^). Additionally, the possible intermolecular magnetic interactions were also investigated for complex 1, as the formation of the supramolecular dimer of dinuclear complexes is found in the solid state by close inspection of the crystal structure. Herein, all interactions were evaluated as listed in Table 2, and the BS-DFT spin densities are shown in Fig. S6.†

**Table 2:** The BS-DFT calculations of the intermolecular isotropic exchange parameters *J*_CuGd_ for 1

	*J* ^R^/*J*^Y^ (cm^−1^) of Cu–Cu′	*J* ^R^/*J*^Y^ (cm^−1^) of Cu–Gd′	*J* ^R^/*J*^Y^ (cm^−1^) of Gd–Gd′
PBE0	−0.33/−0.67	0.09/0.26	−0.01/−0.01
M06-2X	−0.26/−0.52	0.13/0.37	0.02/0.02
TPSSh	−0.23/−0.45	−0.03/−0.09	−0.02/−0.02

The BS-DFT calculations revealed the weak antiferromagnetic exchange for the Cu–Cu′ interactions (*d*(Cu–Cu′) = 6.343 Å) and very weak interactions for Cu–Gd′ of ferromagnetic nature (PBE0 and M06-2X) or antiferromagnetic nature (TPSSh). The Gd–Gd′ interactions seem to be negligible due to the large interatomic distance, *d*(Gd–Gd′) = 9.506 Å. As all reported compounds are isostructural, we can conclude that intermolecular magnetic interactions of similar magnitude are also present in 2–4.

Next, CASSCF calculations are discussed. The single ion properties for 2–4 account for the lack of SMM behaviour in the AC studies. For 2, the ligand field splits the states into singlets, being consistent with the absence of an energy barrier to the relaxation of this system. For 3, a ground doublet with *g*-values *g*_*x*_ = 0.0252, *g*_*y*_ = 0.0427, and *g*_*z*_ = 18.2423 is observed, while the first excited state is found to be at 73 cm^−1^. In the case of 4, *g*-values for the ground doublet are found to be *g*_*x*_ = 1.1647, *g*_*y*_ = 2.9271, and *g*_*z*_ = 14.1239, while the first excited state lies at 35 cm^−1^. The electronic characteristics of Dy^III^ and Er^III^ single ions can be rationalized by considering the electron density of the lanthanide. The largest *m*_*J*_ state for Dy^III^, with an oblate electron density, is best stabilized by an axial ligand field. The *g*-values and separation of the ground state and the first excited state obtained from the CASSCF calculations suggest a slightly axial ligand field for the Dy^III^ ion in 2. In contrast, the *g*-values for the Er^III^ ion are not axial and the wavefunctions are rather mixed, arising due to the prolate electron density of Er^III^, which is better stabilized by an equatorial ligand field. As can be inferred, although the Dy^III^ results show that the ligand field is axial, it does not provide enough axiality to make the system an SMM. In contrast, for Er^III^, the same ligand field is not suited to stabilize an anisotropic ground state.

At this stage, the CASSCF calculations allow us to have insight into the crystal field parameters of the complexes, and hence, it is possible to quantify the interaction operating between the Cu–Ln pairs.^94,95^ Fitting of *χ*_M_*T*(*T*) and *M*(*B*, *T*) was carried out employing the Lines model^83,95^, implemented in PHI,^83^ with the formula:4
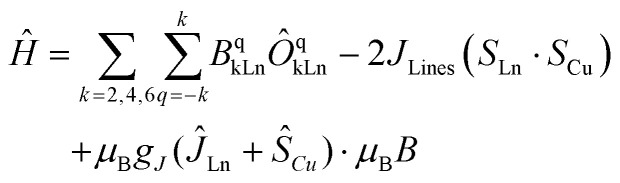
where the first term represents the crystal field parameters, *B*^q^_kLn_, and the operators, *Ô*^q^_kLn_, for the anisotropic lanthanide. The second term represents the Lines interaction connecting the isotropic component of the angular momenta of the lanthanide (*S*_Ln_) and the spin of Cu^II^ (*S* = 1/2) and the last term is the Zeeman term. By simultaneously fitting the *χ*_M_*T*(*T*) and *M*(*B*) experimental data, good agreement is found for 2 with *J*_Lines_ = +3.02(5) cm^−1^ and *zJ* = −0.0046(2) cm^−1^ (solid lines in Fig. 2b). For 3, the following parameters were obtained: *J*_Lines_ = +4.9(1) cm^−1^ and *zJ* = −0.0011(1) cm^−1^, while for 4, the best fit yields *J*_Lines_ = +4.5(1) cm^−1^ and *zJ* = −0.0045(4) cm^−1^ (solid lines in Fig. 2c and d, respectively). Note that clear ferromagnetic interactions are observed in the *χ*_M_*T*(*T*) and *M*(*B*,*T*) experimental data (and by the fitting), and through the fitting of the *χ*_M_*T*(*T*) and *M*(*B*,*T*) profiles, indicating the existence of ferromagnetic coupling in all Cu–Ln systems herein described. The Lines exchange can be projected onto an *m*_*J*_ = 6 state for Tb^III^ and an *m*_*J*_ = 15/2 state for Dy^III^ and Er^III^ leading to *J*^CuTb^_Lines_ = +1.51(1) cm^−1^, *J*^CuDy^_Lines_ = +1.63(1) cm^−1^ and *J*^CuEr^_Lines_ = +0.9(1) cm^−1^, similar to the ones observed for other reported systems.^93^

With the full picture at hand (*i.e.*, magnetic data and CASSCF calculations), we can understand the dynamic magnetic properties of 2–4. Magnetic AC susceptibility measurements show no SMM behaviour for any of the complexes, while μ-SQUID loops show a very narrow opening for 2 and 3. In contrast, a larger opening of the loops is observed for 4 (up to near ∼1 K). CASSCF calculations for the single-lanthanide ions show that the ground states are rather scrambled (3 and 4) and/or with a relatively small separation between the ground doublet and the first excited state (2 and 4). The lack of SMM characteristics can therefore be ascribed to the non-Ising nature of the lanthanide ions. The μ-SQUID loops for 2 and 3, besides displaying rather narrow loops, also highlight the existence of antiferromagnetic coupling, which can be ascribed to the intermolecular interactions. Since no out-of-phase component was observed for 4 in the AC studies and the CASSCF results show that the Er^III^ ion also is characterized by a non-Ising doublet, 4 could not be an SMM. Therefore, the opening of the loop observed for 4 could be of intermolecular ordering and not of single-molecule origin. This fact can also be supported by the ferromagnetic intermolecular interaction obtained by simultaneous fitting of the *χ*_M_*T*(*T*) and *M*(*B*) data (*vide supra*). Our findings in terms of the nature and magnitude of the 3d/4f interactions are in good agreement with previous reports.^37,53,85,93^ Unfortunately, the complexity of the systems, *i.e.*, ligand field, geometry, and intra/intermolecular interactions, and the highly sensitive nature of the relaxation dynamics of the lanthanides preclude further magneto-structural correlations.

## Conclusions

In the present work, we have synthesized four new heterometallic Cu⋯Ln complexes from an *in situ* reaction of a compartmental ligand and the corresponding lanthanide nitrate salts, yielding four isostructural and isomorphous systems. We find the geometry of the Cu^II^ centre to be square pyramidal, while Ln^III^ possesses a muffin-like geometry with *C*_s_ symmetry. Magnetic studies show a ferromagnetic coupling between Cu^II^ and its respective Ln^III^ ions in all complexes. Single crystal sub-Kelvin μ-SQUID magnetic measurements highlight that complexes 1–3 show fast relaxation, while a slow relaxation process is observed for complex 4. Furthermore, in complexes 1–3, the presence of a weak antiferromagnetic coupling is directly observed as a plateau near zero field in the *M*(*B*) curves measured by μ-SQUID. Simulation of *χ*_M_*T*(*T*) and *M*(*B*, *T*) allows us to assess the magnitude and nature of the interactions operating in the complexes. We find an overall ferromagnetic intramolecular interaction in all systems and a weak intermolecular antiferromagnetic interaction. The antiferromagnetic coupling can be explained by considering the intermolecular Cu⋯Cu distance in the crystal structure, as supported by BS-DFT calculations. Finally, CASSCF calculations for the single-lanthanide ions showed that the ground states are rather scrambled (3 and 4) and/or with a relatively small separation between the ground doublet and the first excited state (2 and 4). The lack of SMM characteristics can therefore be ascribed to the non-Ising nature of the lanthanide ions, and further modification of the ligand environment or exchange of the 3d metal ion may prevent it. In this work, we show a general route by which interactions in 3d–4f complexes can be revealed through SQUID, μ-SQUID magnetometry, BS-DFT and CASSCF calculations.

## Author contributions

A. P. conceived and designed the project and also conducted the synthesis of the complexes together with the analysis and interpretation of X-ray crystallographic data. N. J. performed general characterization studies of the compounds. P. B. collected and processed single-crystal X-ray diffraction data. S. P. and W. W. collected, processed, and interpreted the μ-SQUID results. G. N. collected and processed the SQUID data. R. H. carried out the DFT calculations and data interpretation. E. M.-P. carried out the CASSCF calculations and performed the SQUID data simulations. The manuscript was written by A. P., S. P. and E. M. -P. with input from all authors. All authors have approved the final version of the manuscript.

## Conflicts of interest

The authors declare no competing financial interests.

## Supplementary Material

DT-053-D3DT03557C-s001

DT-053-D3DT03557C-s002
