# Supplementary material for: Insight into ferromagnetic interactions in CuII–LnIII dimers with a compartmental ligand
Source: Dalton Trans. 2023 Dec 27;53(6):2501–11. doi: 10.1039/d3dt03557c (PMC10845014; doi:10.1039/d3dt03557c)
Supplement: DT-053-D3DT03557C-s001 [file DT-053-D3DT03557C-s001.pdf]

Electronic Supplementary Material (ESI) for Dalton Transactions.
This journal is © The Royal Society of Chemistry 2023

Supplementary information

Anangamohan Panja[a,b,*], Sagar Paul[c,*], Eufemio Moreno-Pineda[d,e], Radovan Herchel,[f] Narayan Ch. Jana[b], Paula Brandão[g], Ghenadie Novitchi[h] and Wolfgang Wernsdorfer[c,i *]

[a] *Department of Chemistry, Gokhale Memorial Girls' College, 1/1 Harish Mukherjee Road, Kolkata-700020, India. E-mail: ampanja@yahoo.co.in*

[b] *Department of Chemistry, Panskura Banamali College, Panskura RS, WB 721152, India*

[c] *Physikalisches Institut, Karlsruhe Institute of Technology, D-76131 Karlsruhe, Germany. E-mail: wolfgang.wernsdorfer@kit.edu*

[d] *Universidad de Panamá, Facultad de Ciencias Naturales, Exactas y Tecnología, Depto. de Química-Física, 0824 Panamá, Panamá.*

[e] *Universidad de Panamá, Facultad de Ciencias Naturales, Exactas y Tecnología, Grupo de Investigación de Materiales, 0824 Panamá, Panamá.*

[f] *Department of Inorganic Chemistry, Faculty of Science, Palacký University, 17. listopadu 12, 77146 Olomouc, Czech Republic*

[g] *Department of Chemistry, CICECO-Aveiro Institute of Materials, University of Aveiro, 3810-193 Aveiro, Portugal*

[h] *Laboratoire National des Champs Magnétiques Intenses, UPR CNRS 3228, Université Grenoble-Alpes, B.P. 166, 38042 Grenoble Cedex 9, France*

[i] *Institute for Quantum Materials and Technology (IQMT), Karlsruhe Institute of Technology (KIT), Eggenstein-Leopoldshafen D-76344, Germany; Physikalisches Institut, Karlsruhe Institute of Technology, Karlsruhe D-76131, Germany*

Table of Contents

Section 1: Structural information 3

Figure S1. IR spectra of 1-4..3

Figure S2. Simulated and experimental powder X-ray pattern of **1-4**..3

Figure S3. Crystal structures of complexes **1, 2** and **4** showing ellipsoid (30 % probability) plots together with the local coordination geometry around the Dy(III) center..4

Figure S4. A representative packing diagram of supramolecular dimer of **3** showing $\pi-\pi$ interaction. ..4

Table S1. Crystallographic data and structure refinement parameters of 1−4. 5

Table S2. Bond distances around metal coordination spheres in **1–4.** 6

Table S3. SHAPE analysis of Cu^{II} ion in complexes **1–4**..6

Table S4. SHAPE analysis of the Ln^{III} ion in complexes **1–4**. 7

Section 2: DFT calculations : 8

Figure S5. The BS-DFT calculated spin density distribution using PBE0 functional for dinuclear complexes of 1-4. The spin densities are represented by yellow/cyan surfaces calculated with a cutoff value of 0.01 e $bohr^{-3}$. Hydrogen atoms were omitted for clarity..8

Figure S6. The BS-DFT calculated spin density distribution using PBE0 functional for the supramolecular dimer of dinuclear complexes of 1. The spin densities are represented by yellow/cyan surfaces calculated with a cutoff value of 0.01 e $bohr^{-3}$. Hydrogen atoms were omitted for clarity. Note: BS-DFT calculations were performed in such a way that only two metal ions were paramagnetic, and two other metals were replaced by diamagnetic ions (Gd→Lu, Cu→Zn). 9

Section 3: CASSCF simulation details..10

Table S5: CASSCF calculated ligand field Bkq parameters for the Ln ion in **CuLn** systems. 10

Table S6: Computed energy levels (the ground state is set at zero) composition of the g-tensor (g_x, g_y, g_z) and the main components (>10%) of the wavefunction for each m_j state of the ground-state multiplet $^6H_{15/2}$ for the Dy ion in **CuDy** at the CASSCF level..10

Table S7: Computed energy levels (the ground state is set at zero) composition of the g-tensor (g_x, g_y, g_z) and the main components (>10%) of the wavefunction for each m_j state of the ground-state multiplet $^4I_{15/2}$ for the Er ion in **CuEr** at the CASSCF level. 11

Section 4: Derivative-field angle map in complex 2 (CuTb) 11

Figure S7. Derivative-field angle map (in complex **2**) obtained from angle dependent M(B) loops measured by a μ-SQUID. 11

Section 1: Structural information

This section consists of structural information of complex **1-4.** Figure S1, S2 shows experimental IR and XRD spectra of the four complexes. Figure S3, S4 show the crystal structure of **1**, **2**, **4** and crystal packing diagram of **3** respectively. Table S1-S4 show crystallographic data, bond distances and shape analysis of these complexes.

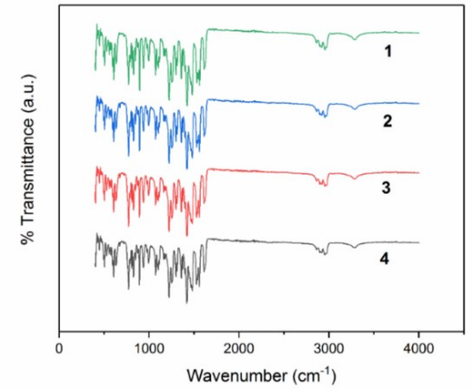

Figure S1. IR spectra of 1-4

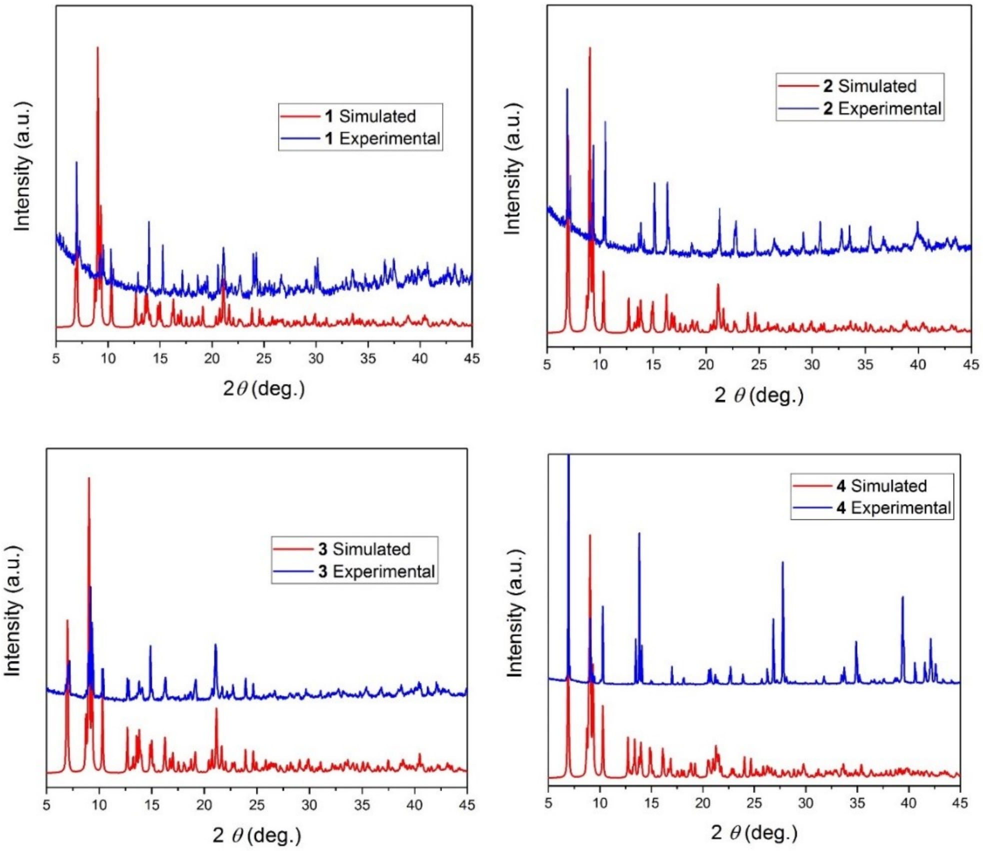

Figure S2. Simulated and experimental powder X-ray pattern of **1-4**.

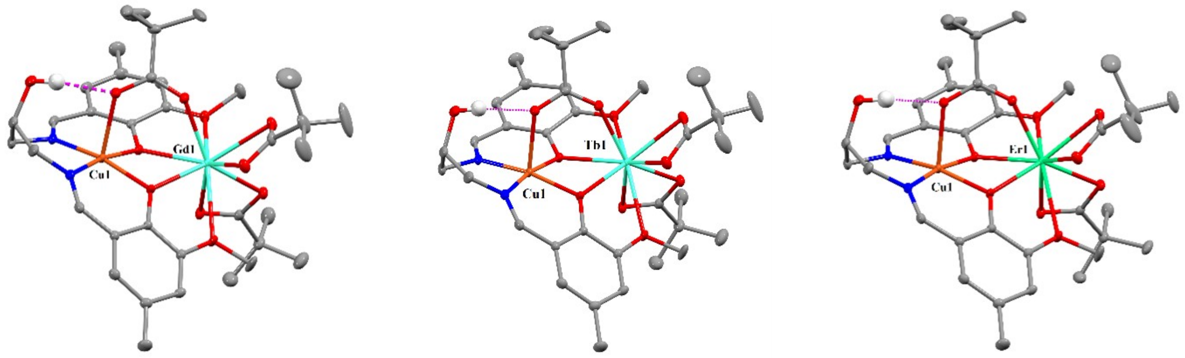

Figure S3. Crystal structures of complexes **1, 2** and **4** showing ellipsoid (30 % probability) plots together with the local coordination geometry around the Dy(III) center

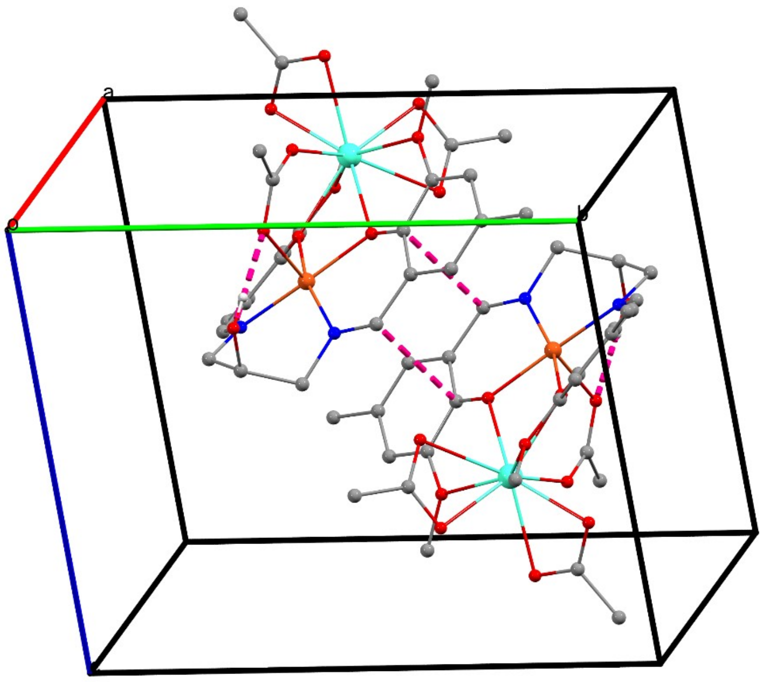

Figure S4. A representative packing diagram of supramolecular dimer of **3** showing π−π interaction. Methyl groups of pivalate ions and most of hydrogen atoms have been omitted for clarity

Table S1. Crystallographic data and structure refinement parameters of 1−4.

	**1**	**2**	**3**	**4**
Empirical formula	$C_{36}H_{51}N_2O_{11}CuGd$	$C_{36}H_{51}N_2O_{11}CuTb$	$C_{36}H_{51}N_2O_{11}CuDy$	$C_{36}H_{51}N_2O_{11}CuEr$
Formula weight	908.57	910.24	913.82	918.58
Temperature/K	150(2)	150(2)	150(2)	150(2)
Crystal system	triclinic	triclinic	triclinic	triclinic
Space group	$P\bar{1}$	$P\bar{1}$	$P\bar{1}$	$P\bar{1}$
a/Å	11.6842(6)	11.6751(7)	11.6733(8)	11.6842(12)
b/Å	13.9182(8)	13.8406(9)	13.8531(10)	13.6822(14)
c/Å	13.9359(8)	13.9768(9)	13.9469(10)	14.1186(13)
α/°	75.986(2)	76.085(3)	76.134(2)	86.239(3)
β/°	65.330(2)	65.374(2)	65.370(2)	65.790(3)
γ/°	68.306(2)	68.336(2)	68.404(2)	68.523(3)
Volume/Å^3	1903.19(19)	1898.0(2)	1896.3(2)	1906.1(3)
Z	2	2	2	2
ρ_{calc} g/cm^3	1.584	1.591	1.600	1.600
μ/mm^{-1}	2.346	2.468	2.576	2.804
F(000)	922.0	924.0	928.0	932.0
2Θ range for data collection/°	4.028 to 58.528	4.146 to 53.004	4.028 to 52.878	5.968 to 58.372
Reflections collected	101308	34319	32693	126257
Independent reflections	10311 [R_{int} = 0.0397, R_{sigma} = 0.0223]	7847 [R_{int} = 0.0717, R_{sigma} = 0.0604]	7783 [R_{int} = 0.0436, R_{sigma} = 0.0438]	10258 [R_{int} = 0.0286, R_{sigma} = 0.0122]
Data/restraints/parameter	10311/0/477	7847/6/487	7783/0/480	10258/2/501
Goodness-of-fit on F^2	1.085	1.067	1.029	1.065
Final R indexes [I>=2σ (I)]	R_1 = 0.0238, wR_2 = 0.0558	R_1 = 0.0442, wR_2 = 0.1096	R_1 = 0.0307, wR_2 = 0.0696	R_1 = 0.0144, wR_2 = 0.0377
Final R indexes [all data]	R_1 = 0.0294, wR_2 = 0.0577	R_1 = 0.0600, wR_2 = 0.1188	R_1 = 0.0422, wR_2 = 0.0736	R_1 = 0.0150, wR_2 = 0.0380
Largest diff. peak/hole / e Å^{-3}	0.82/-0.59	1.92/-1.70	1.08/-0.73	0.83/-0.56

Table S2. Bond distances around metal coordination spheres in **1−4.**

**Bond**	**Distances**			
	**1**	**2**	**3**	**4**
Ln–O1	2.3384(15)	2.389(3)	3.3969(5)	2.2929(10)
Ln–O2	2.6525(15)	2.505(3)	3.3969(5)	2.6621(10)
Ln–O3	2.4023(15)	2.318(3)	2.375(2)	2.3542(9)
Ln–O4	2.5103(16)	2.654(3)	2.490(2)	2.4745(10)
Ln–O6	2.3645(16)	2.340(3)	2.330(2)	2.3051(10)
Ln–O7	2.4972(16)	2.479(3)	2.466(2)	2.4423(11)
Ln–O8	2.4006(16)	2.387(3)	2.373(2)	2.3502(10)
Ln–O9	2.4105(16)	2.384(3)	2.378(2)	2.4369(10)
Ln–O10	2.4719(16)	2.461(3)	2.449(2)	2.3511(11)
Cu–O1	1.9444(15)	1.942(3	1.945(2)	1.9468(10)
Cu–O3	1.9433(15)	1.954(3)	1.941(2)	1.9373(10)
Cu–N1	1.988(2)	2.215(3)	1.977(3)	1.9839(13)
Cu–N2	1.9684(19)	1.988(4)	1.970(3)	1.9708(12)
Cu–O5	2.2164(16)	2.215(3)	2.215(3)	2.2114(10)
Ln···Cu	3.4206(3)	3.4050(8)	3.3969(5)	3.3758(3)

Table S3. SHAPE analysis of Cu^{II} ion in complexes **1**–**4**.

Label	Shape	Symmetry	**1**	**2**	**3**	**4**
PP-5	Pentagon	D_{5h}	29.472	29.450	29.416	29.588
vOC-5	Vacant octahedron	C_{4v}	1.319	1.301	1.339	1.339
TBPY-5	Trigonal bipyramid	D_{3h}	4.844	4.873	4.920	4.905
SPY-5	Spherical square pyramid	C_{4v}	1.105	1.114	1.148	1.146
JTBPY-5	Johnson trigonal bipyramid J12	D_{3h}	7.465	7.517	7.574	7.554

Table S4. SHAPE analysis of the Ln^{III} ion in complexes **1–4**.

Label	Shape	Symmetry	**1** (Gd)	**2** (Tb)	**3** (Dy)	**4** (Er)
EP-9	Enneagon	D_{9h}	35.478	35.364	35.350	35.025
OPY-9	Octagonal pyramid	C_{8v}	23.211	23.203	23.221	23.381
HBPY-9	Heptagonal bipyramid	D_{7h}	17.019	17.064	17.039	17.165
JTC-9	Johnson triangular cupola J3	C_{3v}	13.282	13.338	13.390	13.332
JCCU-9	Capped cube J8	C_{4v}	9.742	9.766	9.770	9.672
CCU-9	Spherical-relaxed capped cube	C_{4v}	8.270	8.330	8.336	8.306
JCSAPR-9	Capped square antiprism J10	C_{4v}	3.415	3.345	3.338	3.210
CSAPR-9	Spherical capped square antiprism	C_{4v}	2.310	2.267	2.260	2.140
JTCTPR-9	Tricapped trigonal prism J51	D_{3h}	3.848	3.701	3.652	3.389
TCTPR-9	Spherical tricapped trigonal prism	D_{3h}	2.630	2.556	2.520	2.408
JTDIC-9	Tridiminished icosahedron J63	C_{3v}	11.468	11.591	11.657	11.931
HH-9	Hula-hoop	C_{2v}	10.825	10.925	10.912	10.997
MFF-9	**Muffin**	$\boldsymbol{C_s}$	**2.005**	**1.988**	**1.989**	**1.992**

Section 2: DFT calculations:

The BS-DFT calculated spin density distributions are shown in the Figures S5 and S6 indicating intra and inter molecular scenario respectively.

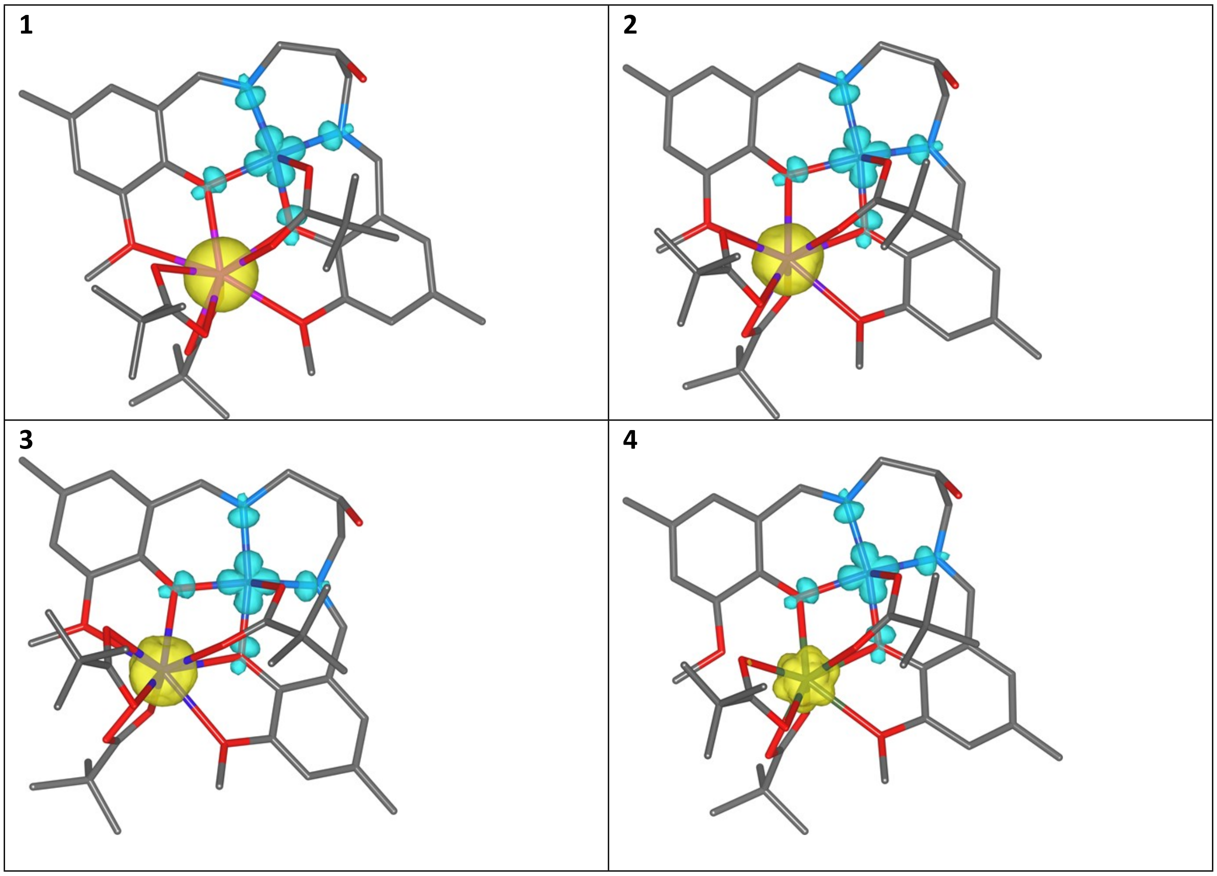

Figure S5. The BS-DFT calculated spin density distribution using PBE0 functional for dinuclear complexes of 1-4. The spin densities are represented by yellow/cyan surfaces calculated with a cutoff value of 0.01 e bohr^{-3}. Hydrogen atoms were omitted for clarity.

Cu-Cu’	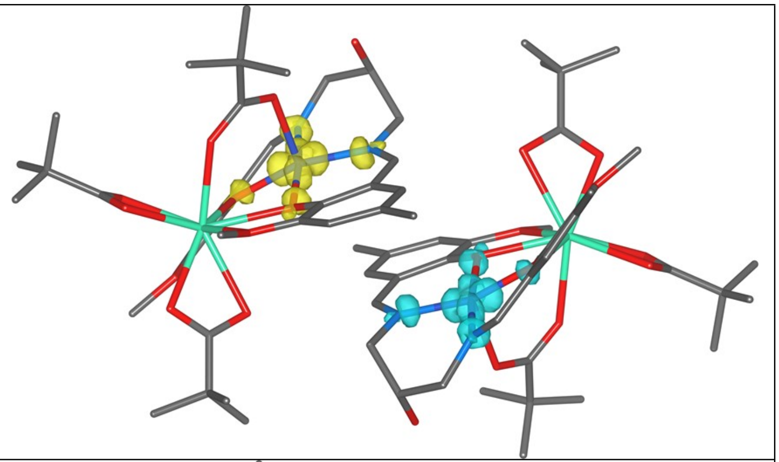
Cu-Gd’	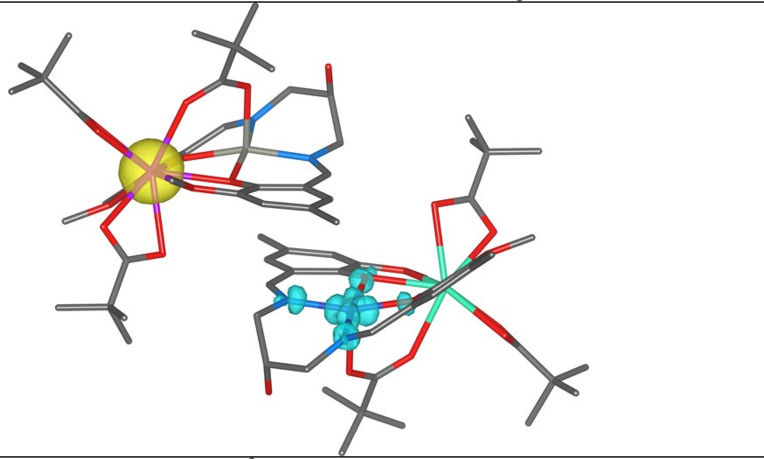
Gd-Gd’	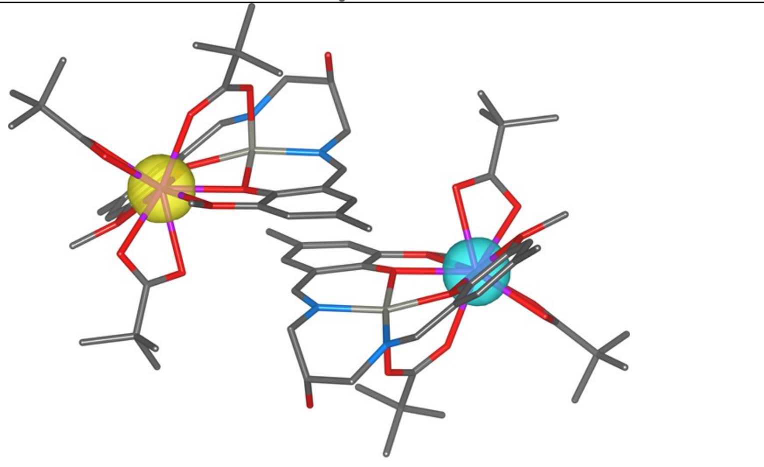

Figure S6. The BS-DFT calculated spin density distribution using PBE0 functional for the supramolecular dimer of dinuclear complexes of 1. The spin densities are represented by yellow/cyan surfaces calculated with a cutoff value of 0.01 e bohr^{-3}. Hydrogen atoms were omitted for clarity. Note: BS-DFT calculations were performed in such a way that only two metal ions were paramagnetic, and two other metals were replaced by diamagnetic ions (Gd→Lu, Cu→Zn).

Section 3: CASSCF simulation details

Ligand field parameters obtained from CASSCF simulations are tabulated in Table S5, while computed energy levels, g values and the wavefunctions for each m_j state are shown in Table S6 and S7 for CuDy and CuEr complex respectively.

Table S5: CASSCF calculated ligand field B_k^q parameters for the Ln ion in **CuLn** systems.

k	*q*	*B(k,q) / Tb*	*B(k,q) / Dy*	*B(k,q) / Er*
2	-2	-1.29942	0.11242	0.10119
2	-1	-0.15729	0.25614	1.74546
2	0	-2.36779	-1.42409	-0.86491
2	1	-1.91969	0.2733	0.03235
2	2	4.7586	2.99981	-0.08627
4	-4	-0.01586	0.01367	-0.01014
4	-3	-0.03091	-0.0113	-0.00752
4	-2	-0.02162	0.01527	-0.00322
4	-1	0.00866	-0.00703	0.00536
4	0	-0.00266	5.02644E-4	-9.23995E-5
4	1	0.01968	0.0012	-0.00576
4	2	-0.0135	-0.0016	-9.5667E-4
4	3	0.07572	0.00343	0.03179
4	4	-0.02161	0.00456	-0.00414
6	-6	-4.84008E-5	9.50413E-5	1.59784E-4
6	-5	-3.65839E-4	8.35451E-5	7.08004E-4
6	-4	-9.39626E-5	-1.11914E-5	-1.18922E-4
6	-3	-1.37852E-4	-1.04334E-4	-2.01353E-4
6	-2	3.80063E-5	8.08437E-5	-9.32596E-5
6	-1	-2.29428E-4	-6.56598E-5	-3.98917E-4
6	0	9.01088E-6	-7.42457E-7	-3.09847E-5
6	1	2.70547E-4	-1.04102E-4	2.0509E-5
6	2	-1.79268E-4	3.14087E-4	-2.0291E-4
6	3	-4.0726E-4	1.63451E-4	-2.04984E-4
6	4	3.29518E-5	-4.96579E-5	-4.36065E-5
6	5	4.77047E-4	-1.33681E-4	7.89623E-4
6	6	-1.26735E-4	4.00246E-5	-4.96148E-4

Table S6: Computed energy levels (the ground state is set at zero) composition of the g-tensor (g_x, g_y, g_z) and the main components (>10%) of the wavefunction for each m_j state of the ground-state multiplet $^6H_{15/2}$ for the Dy ion in **CuDy** at the CASSCF level.

***Energy* (cm^{-1})**	g_x	g_y	g_z	***Wavefunction***
0	0.0252	0.0427	18.2423	68% |±15/2> + 28% |±11/2>
73.0	0.4359	0.9219	14.8378	59% |±13/2> + 22% |±9/2>
114.8	0.0072	1.3945	16.7622	11% |±5/2> + 27% |±1/2>
195.4	4.0781	6.1394	10.7624	29% |±11/2> + 26% |±7/2> + 11% |±5/2>
222.1	0.1329	4.2544	11.1130	16% |±11/2> + 15% |±9/2> + 12% |±7/2>
264.2	1.9653	2.8447	14.1885	15% |±11/2> + 20% |±9/2> + 10% |±7/2>
311.9	0.8042	0.9839	16.6783	21% |±7/2> + 29% |±5/2> + 20% |±3/2>
491.9	0.0055	0.0140	19.8298	15 |±5/2> + 43% |±1/2> + 31% |±3/2>

Table S7: Computed energy levels (the ground state is set at zero) composition of the g-tensor (g_x, g_y, g_z) and the main components (>10%) of the wavefunction for each m_j state of the ground-state multiplet $^4I_{15/2}$ for the Er ion in **CuEr** at the CASSCF level.

Energy (cm^{-1})	g_x	g_y	g_z	*Wavefunction*
0	1.1647	2.9271	14.1239	57% |±15/2> + 20% |±11/2>
34.6	0.4313	2.2900	13.2683	23% |±15/2> + 25% |±9/2> + 10 |±7/2>
92.0	0.7777	1.5643	12.6179	23% |±13/2> + 14% |±9/2> + 22 |±7/2>
128.1	2.2953	6.3282	8.7334	22% |±13/2> + 12% |±5/2> + 17 |±3/2>
190.7	1.2567	3.4086	8.7992	14% |±11/2> + 21% |±9/2> + 11 |±5/2> + 13 |±1/2>
211.0	0.6385	1.6969	7.6003	12% |±13/2> + 10% |±7/2> + 14 |±5/2> + 16 |±3/2>
268.4	3.1063	4.3425	6.9089	19% |±11/2> + 14% |±7/2> + 18 |±5/2> + 20 |±3/2>
328.5	1.0319	2.5268	13.3377	15% |±11/2> + 15% |±9/2> + 10 |±3/2> + 30 |±1/2>

Section 4: Derivative-field angle map in complex 2 (CuTb)

The μ-SQUID measurement setup equipped with 3D vector magnet allows angle dependent $M(B)$ with an angular precision better than 0.1° without manually rotating the sample. However, the external field direction is confined within the μ-SQUID plane to avoid flux due to the external field and measure magnetic signal coming only from the single crystal. Figure S7 shows derivative dM/dB mapped with direction of applied field (B_x, B_y) as derived from experimental M(B) curves (positive cycle) measured at different angles of external field. The observed parallel lines indicate the switching fields between antiferromagnetic (AFM) to ferromagnetic (FM) state. Only one pair of lines confirm a single easy axis for the FM/AFM ordering.

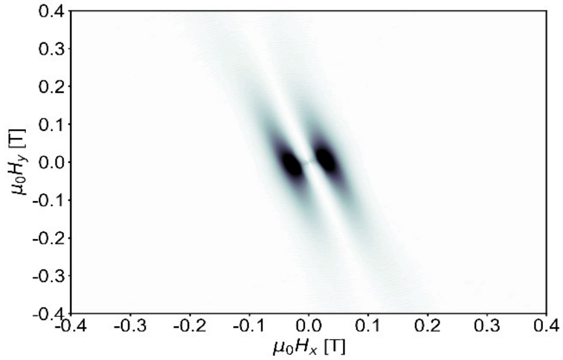

Figure S7. Derivative-field angle map (in complex **2**) obtained from angle dependent M(B) loops measured by a μ-SQUID.
